# Prediction model for COVID-19 patient visits in the ambulatory setting

**DOI:** 10.21203/rs.3.rs-177379/v1

**Published:** 2021-03-02

**Authors:** Riza C. Li, Cecelia K. Harrison, Claudine T. Jurkovitz, Mia A. Papas, Kevin Ndura, Roger Kerzner, Cydney Teal, Tze Chiam

**Affiliations:** Christiana Care Health System; Christiana Care Health System; Christiana Care Health System; Christiana Care Health System; Christiana Care Health System; Christiana Care Health System; Christiana Care Health System; Christiana Care Health System

**Keywords:** Ambulatory, COVID-19, ARIMA, time series analysis, family medicine

## Abstract

**Objective:**

Healthcare systems globally were shocked by coronavirus disease 2019 (COVID-19). Policies put in place to curb the tide of the pandemic resulted in a decrease of patient volumes throughout the ambulatory system. The future implications of COVID-19 in healthcare are still unknown, specifically the continued impact on the ambulatory landscape. The primary objective of this study is to accurately forecast the number of COVID-19 and non-COVID-19 weekly visits in primary care practices.

**Materials and Methods:**

This retrospective study was conducted in a single health system in Delaware. All patients’ records were abstracted from our electronic health records system (EHR) from January 1, 2019 to July 25, 2020. Patient demographics and comorbidities were compared using t-tests, Chi square, and Mann Whitney U analyses as appropriate. ARIMA time series models were developed to provide an 8-week future forecast for two ambulatory practices (AmbP) and compare it to a naïve moving average approach.

**Results:**

Among the 271,530 patients considered during this study period, 4,195 patients (1.5%) were identified as COVID-19 patients. The best fitting ARIMA models for the two AmbP are as follows: AmbP1 COVID-19+ ARIMAX(4,0,1), AmbP1 nonCOVID-19 ARIMA(2,0,1), AmbP2 COVID-19+ ARIMAX(1,1,1), and AmbP2 nonCOVID-19 ARIMA(1,0,0).

**Discussion and Conclusion:**

Accurately predicting future patient volumes in the ambulatory setting is essential for resource planning and developing safety guidelines. Our findings show that a time series model that accounts for the number of positive COVID-19 patients delivers better performance than a moving average approach for predicting weekly ambulatory patient volumes in a short-term period.

## Introduction

Healthcare systems were globally shocked by a novel coronavirus, severe acute respiratory syndrome coronavirus 2 (SARS-CoV-2) and the resulting disease, coronavirus disease 2019 (COVID-19). ^1–3^ On March 11, 2020, COVID-19 was declared a global pandemic by the World Health Organization (WHO).^4^ By mid-March, transmission of COVID-19 had rapidly accelerated, increasing case counts throughout the United States, and it was found that many patients with severe disease also had common comorbidities such as hypertension, obesity and diabetes.^5,6^ In the state of Delaware, the first presumptive positive case of COVID-19 was reported by the Delaware Division of Public Health on March 11, 2020.^7^ In order to mitigate the spread of the virus, the Governor of Delaware declared a state of emergency on March 13, 2020. The weeks that followed included several modifications to the original state of emergency to minimize the spread of the virus.

In response to the growing pandemic, ChristianaCare Health Services, Inc. (ChristianaCare), which serves the majority catchment area of Northern Delaware and the most populous county in the state, followed suit with its own measures to mitigate spread, postponing all elective procedures in hospitals and all ambulatory practices effective March 17, 2020 to adhere to state and CDC guidelines. The ambulatory services at ChristianaCare adjusted the delivery of healthcare services by reducing the number of in-person visits to minimize the risk to patients and healthcare providers, redirecting patients to telehealth when appropriate. This resulted in a decrease of patient volumes throughout the ambulatory system. With the uncertainty that COVID-19 presented then, the Phase 1 reopening that occurred on June 1, 2020, and the exponential rise in cases occurring now, it is essential to understand how the ambulatory setting will continue to be affected in order to develop proper guidelines.

To understand the impact of the novel virus, scientists rely on community spread models to predict possible transmission. The popular susceptible, infected, and recovered (SIR) epidemiologic model and variations of this model have been used to gauge community spread of a variety of infectious diseases such as influenza and dengue fever.^8–12^ SIR models have also been applied to inpatient settings to predict hospital capacity regarding admissions, ICU beds, and ventilators.^9,11,13,14^ In addition to SIR models, the current literature on predicting patient volume varies from descriptive statistics to advanced time series models, with most of the studies that have used time series forecasting models focusing on emergency department and hospital admissions.

Time series forecasting in ambulatory visits prior to the COVID19 pandemic have been described in a few reports.^15–22^ The most used method for time series forecasting is the Box-Jenkins method otherwise known as the AutoRegressive Integrated Moving Average (ARIMA) model.^23^ The ARIMA model has been used for its simplicity and flexibility in capturing linear patterns in a time series.^17,19–22^

### Significance

The future implications of COVID-19 in healthcare are still unknown, specifically how it will continue to affect the ambulatory landscape. This work aims to inform COVID-19 and nonCOVID-19 ambulatory resources allocation as well as guide ambulatory practices reopening for in-person visits as in-person care might have been delayed. We propose an ARIMA time series model to capture the changes in ambulatory patient volumes as a result of COVID-19.

### Objective

The primary objective of this study is to accurately forecast the number of COVID-19 and nonCOVID-19 weekly visits in primary care practices. The ability to forecast patient volumes in primary care locations by accurately evaluating the dynamic changes in patient visits and fitting these data to a statistical model is useful for the appropriate allocation of human and material resources for future planning. With the uncertainty that COVID-19 presents, healthcare systems have been adapting their ambulatory practices to adhere to state guidelines and prepare for state reopening phases. Therefore, we developed a time series model that provides an 8-week future forecast for ambulatory practices and compared it to a naive moving average approach.

## Materials And Methods

### Study Design

This retrospective study was conducted in a single health care system in Delaware (ChristianaCare), serving the primary catchment area of New Castle County. New Castle County is in the northernmost region of Delaware and as of 2019 has an estimated population of 558,753, accounting for nearly two-third of the entire state population.^24^ We selected the patients’ records from the two practices that had the highest historical patient volumes among all clinics affiliated with ChristianaCare. Our study population included (1) COVID-19 patients who had prior family medicine ambulatory services within ChristianaCare in 2019 and had been previously hospitalized and discharged or were self-monitoring at home and had not been hospitalized for the disease. (2) Any patient who utilized ambulatory services from the same practices during the same time period and were not diagnosed with COVID-19. COVID-19 patients currently hospitalized were excluded from the population.

We extracted all patients’ records from our electronic health records system (EHR) from January 1, 2019 to July 25, 2020 and built two datasets. One included patient-level data (e.g. age, gender, race, ethnicity, insurance, marital status, and Elixhauser comorbidities and the other ambulatory practice-related data (e.g. encounter location, encounter providers, and weekly patient volumes).^25^ Patient-level data were used for characterizing the study population. Ambulatory practice-related data were primarily used for our time series models. For model development, we used one year of data between January 2019 and December 2019, and for model validation data from January 2020 until the most recent data available (July 2020).

### Statistical and Forecasting Methods

#### Descriptive statistics

Patient demographics and comorbidities were compared using t-tests, Chi square, Mann Whitney U analyses as appropriate according to the distribution.

#### Time-series

A time series is a sequential set of data points, measured typically over successive times. The ARIMA model was created for auto-correlated and non-stationary time series data.^11^ The framework for ARIMA is displayed in Table 1.

Most patients were seen by clinicians in the Department of Family Medicine, with 28% of COVID-19 patients and 26% of non-COVID-19 patients primarily utilizing family medicine services. The forecasting method used is a non-seasonal ARIMA, and ARIMA with exogenous variables (ARIMAX) to predict COVID-19 and nonCOVID-19 weekly patient volumes for Ambulatory Practice 1 (AmbP1) and Ambulatory Practice 2 (AmbP2). The COVID-19 models included an exogenous variable, the weekly number of positive COVID-19 patients, and were significant at p-value < 0.05. When incorporating the exogenous variable to the nonCOVID-19 models, we found them to be insignificant. The complete dataset was split, 75:25 for training and validation sets. The individual models were trained on weekly patient volume data from each location and validated on the most up-to-date 2020 practice volume data available at the time of model development. The validity of our models was evaluated using the difference between the forecasted patient volume and the actual patient volume beyond the period on which the model was trained. For each model out-of-sample forecast errors were assessed by calculating the Root Mean Square Error (RMSE) and mean absolute percentage errors (MAPE) as follows:

(1) $$RMSE=\sqrt{\frac{1}{n}\sum_{i=1}^{n}{\left(y_{i}-\hat{y_{i}}\right)}^{2}}$$

(2) $$MAPE=\left(\frac{1}{n}\sum_{i=1}^{n}\left|\frac{y_{i}-{\hat{y}}_{i}}{y_{i}}\right|\right)*100$$

Where ***n*** represents the total data points, ***y***_***i***_ represents the observed values at time ***i*** and <mi> represents the forecasted value at time ***i.*** Once we evaluated the models performance by computing the RMSE and MAPE, the (***p,d,q***) parameters was used to forecast the next 8-week patient volumes for both populations in each location.

The model selection was performed using the Python 3.8.3 software package *statsmodels.*

### Ethics Approval

The protocol was approved by Expedited Review by the Christiana Care Health System Institutional review board (IRB). Informed consent was waived by Christiana Care IRB in accordance with the Office for Human Research Protections (OHRP) regulations 45 CFR 46.116(d).

## Results

Among the 271,530 patients considered during this study period, 4,195 patients (1.5%) were identified as COVID-19 patients. The COVID-19 patients were younger, and majority were non-white compared to the COVID-19 negative patients. Both COVID-19 positive and negative patients had multiple comorbidities such as obesity, diabetes and hypertension despite the COVID-19 patients being younger. Details of the patient demographics and comorbidities for the study population are provided in Tables 2 and 3.

## Discussion

The novelty of the COVID-19 virus created a conundrum, not only for the inpatient world, but for the ambulatory outpatient clinical environment as well. While many people were being hospitalized due to complications from the virus, an overwhelming majority were being evaluated and treated in the outpatient setting, either by urgent care or by their primary care provider. At the height of the pandemic, prediction models only existed for hospitals to anticipate the need for staffing, personal protective equipment (PPE), equipment and other resources as the cases surged, which made it very challenging to anticipate staffing, equipment and logistical needs for primary care practices. There were many questions/scenarios to consider such as designating specific practices to care for COVID-19 infected patients, estimating the number of staffing and PPE necessary at each practice site for in person care vs delivering telehealth care; redeployment of staff to our Ambulatory COVID-19 treatment center (in person care for non-emergent patients with COVID-19) and to our Virtual COVID-19 primary care practice, which monitored moderately ill patients infected by the disease via video visits and secure texting, based on ambulatory patient volumes. Development of the ambulatory COVID-19 model provided the opportunity to identify volume trends and anticipate the need to modify our care delivery models based on the estimates.

We found that the ARIMA/ARIMAX forecasting models considered in this study outperformed more traditional modeling approaches such as the moving average-based approach. Although a MAPE of < 10% is considered an accurate forecast, the COVID-19 ARIMA models we generated provided a more dynamic prediction than the moving average forecast. Our model accounted for the number of weekly positive COVID-19 cases.

Our findings support the use of such models by health care system administrators to forecast patient volumes and make prospective resource adjustments.

Data utilized for the test set prediction included the time period from March 1, 2020 – July 25, 2020 and was not inclusive of the months during which flu season typically occurs. The impending flu season brings much uncertainty in relation to COVID-19 for both inpatient and ambulatory settings because the two infections may overlap in the winter season. COVID-19 and the flu share clinical characteristics, therefore differentiating between the illnesses is paramount.^26^ This convergence has the potential to yet again overwhelm healthcare systems especially the ambulatory environment, which is often the first point of contact during respiratory virus season. It is thought that the possible resurgence of COVID-19 would lead to tighter social distancing measures which would lessen flu transmission.^27^ However, in absence of that certainty, having an ambulatory prediction model to project patient volumes is crucial.

### Limitations

The current study is limited to retrospectively using electronic health records and positive COVID-19 results from only one hospital system. Our results may not be generalizable to other hospital systems, particularly those who serve patients with different characteristics. Other forecasting methods may be appropriate for different hospitals due to the differences in organizational structure and resources. Our study period is limited to one year of historical data, ignoring potential factors such weather and seasonal affects that could possibly improve forecasting accuracy. However, due to the unpredictable nature of COVID-19 our regular volumes and trends were disrupted. Therefore, using volumes from more years might not actually give us any more accurate prediction since our system is in a transient state, especially during the time of this study. Also, our weekly predictions did not differentiate between in-person and virtual volumes. In future studies, dividing the volumes between in-person and virtual could improve accuracy and provide additional information to healthcare providers for resource planning. Lastly, our models are short-term forecasts. Long-term forecasts can be generated, although the error rate will increase as the prediction period increases.

## Conclusion

Accurately predicting future patient volumes in the ambulatory setting is essential for resource planning and developing guidelines for safely providing appropriate in-person visits. This study contributes to the exploration of time series modeling to forecast ambulatory patient volumes in ChristianaCare during the COVID-19 pandemic. We compared the forecasting accuracy of a moving average approach and ARIMA. Our findings show that a time series model that accounts for the number of positive COVID-19 patients delivers better performance for predicting weekly ambulatory patient volumes in a short-term period. This improved forecasting ability can be used to aid health systems administrators develop guidelines for clinic operations.

## Figures and Tables

**Table 1:** ARIMA framework

**1**	**Visualize the data as a time series**
**2**	Test time series data for stationarity (e.g. Augmented Dickey Fuller Test); if non-stationary, transform the data
**3**	Estimate model parameters (*p,d,q*) (e.g. Autocorrelation Function (ACF), and Partial Autocorrelation Function (PACF)
**4**	Identify best model parameters using fit criteria (e.g. Akaike Information Criterion (AIC) and Bayesian Information Criterion (BIC))
**5**	Apply diagnostic tools to determine model fitness (e.g. Plot of standardized residuals, Histogram plus estimated density, Normal Q-Q plot, and Correlogram plot)
**6**	Forecast *n* weeks using an independent dataset
**7**	Evaluate the accuracy of the forecast using error statistics (e.g. RMSE and MAPE)

**Table 2:** Study Population Characteristics

	nonCOVID-19 n = 267,335	COVID-19 n = 4,195	p value
Age (mean, SD)	48.8 (22.7)	44.9 (19.0)	<0.01
Age category, n (%)			<0.01
<18, n (%)	31515 (11.8)	179 (4.3)	
18–<45, n (%)	75394 (28.2)	1970 (47.0)	
45–<65, n (%)	84435 (31.6)	1394 (33.2)	
≥ 65, n (%)	75991 (28.4)	652 (15.5)	
Male, n (%)	104677 (39.2)	1856 (44.2)	<0.01
Race, n (%)			<0.01
White	185300 (69.3)	1634 (39.0)	
African American	58965 (22.0)	1477 (35.2)	
Asian	9648 (3.6)	81 (1.9)	
Other	8186 (3.1)	564 (13.4)	
Missing	5236 (2.0)	439 (10.5)	
Hispanic, n (%)	13527 (5.1)	896 (21.4)	<0.01
Insurance, n (%)			<0.01
Commercial	150097 (56.2)	2189 (52.2)	
Medicaid	32835 (12.3)	640 (15.3)	
Medicare	71910 (26.9)	645 (15.4)	
Self-Pay	12493 (4.6)	9 (0.2)	
Missing	0 (0.0)	712 (16.9)	
Married, n (%)	124563 (46.6)	1593 (38.0)	<0.01
Have any Comorbidities, n (%)	260920 (97.6)	3666 (87.4)	<0.01

**Table 3:** Study Population Comorbidities

	non COVID 19 n = 267,335	COVID-19 n = 4,195	p value
Comorbidity Count (median, IQR)	2.0 (0.0–4.0)	2.0 (0.0–4.0)	< 0.01
Hypertension, n (%)	104331 (39.0)	1363 (32.5)	< 0.01
Congestive heart failure, n (%)	15790 (5.9)	236 (5.6)	< 0.01
Diabetes, n (%)	42194 (15.8)	741 (17.7)	< 0.01
Liver Disease, n (%)	19082 (7.1)	253 (6.0)	< 0.01
Renal Failure, n (%)	16264 (6.1)	287 (6.8)	< 0.01
Chronic Lung, n (%)	60898 (22.8)	804 (19.2)	< 0.01
Depression, n (%)	62104 (23.2)	748 (17.8)	< 0.01
Obesity, n (%)	70752 (26.4)	1022 (24.4)	< 0.01
Coronary Heart Disease, n (%)	28210 (10.6)	355 (8.5)	< 0.01
Cardiac Arrhythmia, n (%)	50207 (18.8)	763 (18.2)	< 0.01

**Table 4.** Validation Dataset Performance *RMSE; MAPE for each model

		ARIMA Models		Moving Average		
	
		ARIMA (*p,d,q*)	RMSE	MAPE	RMSE	MAPE

AmbP1						
	COVID-19	ARIMAX (4,0,1)	2.22	24.86	2.92	23.21
	
	nonCOVID-19	ARIMA (2,0,1)	67.13	7.83	96.05	13.18

AmbP2						
	COVID-19	ARIMAX (1,1,1)	3.62	14.49	4.73	29.75
	
	nonCOVID-19	ARIMA (1,0,0)	79.46	8.55	86.15	15.53
